# Notes and News

**Published:** 1900-01-06

**Authors:** 


### NOTES AND NEWS.

WE much regret that, following an announcement in the *Times*, we last week gave currency to a report of the death of Mr. Cadge, of Norwich.

ACCORDING to the weekly return issued by the Registrar-General, out of the total of 3,067 deaths registered in London 193 were directly attributed to influenza, the numbers in the preceding three weeks having been 42, 38, and 69 respectively.

THE present position of the vaccination difficulty at Leicester is that the Local Government Board have sent a communication to the Guardians stating that they will sanction the appointment of the vaccination officer who has been chosen, on condition that he is also appointed under the Act of 1898, and consents to carry out the orders of the Local Government Board. This, we understand, the officer has agreed to do.

THE number of consulting surgeons appointed to act in South Africa has still further been increased. Sir William Stokes, surgeon-in-ordinary to the Queen in Ireland; Mr. Watson Cheyne, professor of surgery at King's College, and surgeon to the hospital; Mr. G. L. Cheatle, teacher of practical surgery at the same college, and assistant surgeon at the hospital; and Mr. Kendal Franks, surgeon-in-ordinary to the Lord Lieutenant in Ireland. Mr. Franks is now in South Africa, Sir William Stokes has sailed, and the other two gentlemen will shortly depart.

LORD IVEAGH is about to provide the sick and wounded soldiers in South Africa with an addition of 100 beds to one of the base hospitals fully equipped, and provided with medical service. Five base hospitals are now established, and the work of the Army Medical Service is terribly hard. At home the question of convalescent homes for officers and men is paramount. Practical people will readily realise that great difficulties in organisation would arise if the offers of numerous small establishments were accepted. At the same time definite arrangements are progressing for the welfare of the convalescing soldier. A large building is preparing at Windsor, containing some 80 beds, and seaside branch establishments in connection with it are contemplated. Officers are not to be overlooked, and these the Duke of Abercorn will probably make his special care.

THE emolument of the post of medical officer to the Local Government Board, vacant through the death of Sir Richard Thorne Thorne, is £1,500 per annum.

ACCORDING to the reports from Lorenzo Marques, Sir J. Sivewright's kindness in organising and sending out an ambulance for the use of the Boers has received a cutting rebuff from our amiable enemies. On arrival at Lorenzo Marques the doctors in charge of the ambulance applied to the Transvaal Consul for passports to Pretoria, but they were curtly informed that the Boers were already more than adequately ambulanced, having practically no wounded, and Sir J. Sivewright's offer was rejected without thanks. This looks like a bit of bounce, and is very characteristic of what appears at present to be the ruling feeling in Pretoria.

ACCORDING to the Cape Town correspondent of the *Times*, complaints are being freely made about the lack of organisation which is apparent in the management of the military hospitals at Cape Town. The pressure caused by the arrival of the ambulance trains has, it is said, been too great for the staff engaged. The arrangements for the reception of the officers are held to be defective, and the number of nurses and orderlies insufficient. The statement is also made that the scale of diet allowed for serious cases is too low for healthy young officers wounded only in the extremities. We hope that in time things will shake down a bit, but there is no doubt that there is a certain amount of local jealousy in regard to the extent to which local help in the way of nursing has been declined.

A MOVEMENT has been started amongst the ladies of England to provide a hospital at the base for the Imperial Yeomanry. An influential committee has been formed, with the Princess of Wales as president and the Duchesses of Connaught and York as vice-presidents. The Duchess of Devonshire, the Duchess of Beaufort, the Duchess of Marlborough, the Countess of Warwick, Lady Georgina Curzon, and numerous other well-known ladies have joined the committee. About £30,000 will be required to equip and provide the hospital, of which £8,000 has been promised. The work will be carried out in conjunction with the War Office and with the Red Cross Society. Contributions in money or kind will be acceptable. The hon. secretary is Viscount Curzon, 20, Curzon Street, Mayfair.

IN the list of New Year's Honours several medical names appear. Knighthood has been conferred upon Dr. Lauder Brunton, F.R.S., the well-known physician to St. Bartholomew's Hospital; and upon Surgeon-General F. Hy. Lovell, C.M.G., of Trinidad, another Bart.'s man. Among those to be Companions of the Most Distinguished Order of St. Michael and St. George are Dr. John Pringle, Member of the Privy Council and Legislative Council of the Island of Jamaica; Dr. Patrick Manson, Medical Adviser to the Colonial Office, well known for his researches in regard to the filaria sanguinis hominis, and as the originator of the mosquito theory of malaria; and Dr. Wordsworth Poole, for services as Principal Medical Officer of the West African Frontier Force on the Niger. Dr. Poole is a Cambridge and Guy's man.

